# Bidirectional Communication Between Microglia and Astrocytes in Neuroinflammation

**DOI:** 10.2174/1570159X21666221129121715

**Published:** 2023-08-15

**Authors:** Anup Bhusal, Ruqayya Afridi, Won-Ha Lee, Kyoungho Suk

**Affiliations:** 1 Department of Pharmacology, School of Medicine, Kyungpook National University, Daegu, Republic of Korea;; 2 Department of Biomedical Sciences, School of Medicine, BK21 Plus KNU Creative BioResearch Group, Kyungpook National University, Daegu, Republic of Korea;; 3 School of Life Sciences, BK21 Plus KNU Creative BioResearch Group, Kyungpook National University, Daegu 41566, Republic of Korea;; 4 Brain Science and Engineering Institute, Kyungpook National University, Daegu 41944, Republic of Korea

**Keywords:** Microglia, astrocytes, crosstalk, neuroinflammation, glia, central nervous system

## Abstract

Neuroinflammation is a common feature of diverse nervous system pathologies. In many instances, it begins at an early stage of the disease, paving the way for further exacerbations. The main drivers of neuroinflammation are brain-resident glial cells, such as microglia and astrocytes. Microglia are the primary responders to any insult to the brain parenchyma, translating the signals into diverse molecules. These molecules derived from microglia can regulate the stimuli-dependent reactivity of astrocytes. Once activated, astrocytes in turn, can control microglia phenotypes. Recent evidence indicates that the crosstalk between these glial cells plays an important role in delaying or accelerating neuroinflammation and overall disease progression. To date, various molecules have been recognized as key mediators of the bidirectional communication between microglia and astrocytes. The current review aims to discuss the novel molecules identified recently, which play a critical role in interglial crosstalk, highlighting their therapeutic potential.

## 1 INTRODUCTION

**Fig. (1):**
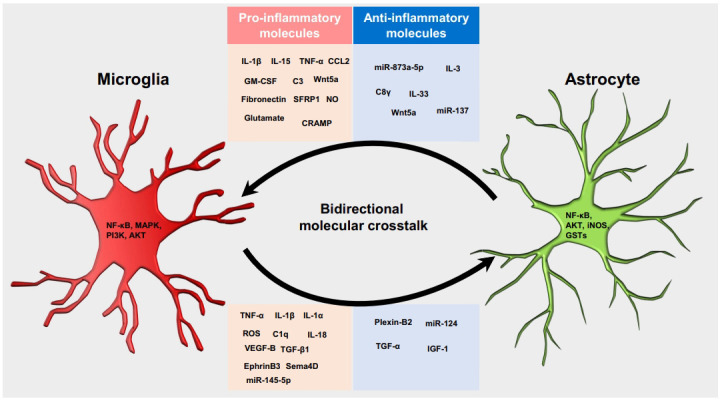
Bidirectional molecular crosstalk between microglia and astrocytes under neuroinflammatory conditions. Microglia and astrocytes secrete context-dependent molecules to regulate each other’s roles in various disease conditions. Microglia can induce the anti-inflammatory/neuroprotective functions of astrocytes in a disease-stage-specific manner by releasing IGF-1, TGF-α, plexin-B2, and miR-124. Conversely, increased inflammatory signaling such as NF-κB, MAPK, PI3K, and Akt can lead to the release of pro-inflammatory mediators, including TNF-α, IL-1α, IL-1β, IL-18, C1q, ROS, Sema4D, EphrinB3, TGF-β, and VEGF-B, from microglia that can potentiate the neuroinflammatory/neurotoxic activities of astrocytes. Similarly, increased NF-κB, iNOS, GSTs, and Akt in astrocytes cause the release of various molecules, such as TNF-α, IL-1β, IL-15, CCL2, C3, GM-CSF, NO, fibronectin, SFRP1, Wnt5a, C8γ, miR-137, IL-3, IL-33, and glutamate, that act on microglia to regulate their functions in health and disease. SFRP1, secreted frizzled-related protein 1; IL, interleukin; CCL2, chemokine (C-C motif) ligand 2; GM-CSF, granulocyte-macrophage colony-stimulating factor; TNF, tumor necrosis factor; NO, nitric oxide; ROS, reactive oxygen species; CRAMP, cathelicidin-related antimicrobial peptide; NF-κB, nuclear factor kappa-light-chain-enhancer of activated B cells; MAPK, mitogen-activated protein kinase; PI3K, phosphoinositide 3-kinases; Akt, protein kinase B; GSTs, glutathione S-transferases; iNOS, inducible nitric oxide synthase; C3, complement component 3; C8γ, complement component 8 gamma; Wnt5a, wingless-type MMTV integration site family member 5a; Sema4D, semaphorin-4D; TGF, transforming growth factor; C1q, complement component 1 q; VEGF-B, vascular growth factor-beta; miR, microRNA; IGF-1, insulin-like growth factor 1.

Neuroinflammation has emerged as a critical regulator of many central nervous system (CNS) pathologies, and it is mediated by complex molecular crosstalk between microglia and astrocytes. Microglia and astrocytes are dynamic glial cells that respond to all CNS insults and undergo context-dependent morphological and functional changes [1]. Both cell types conduct continuous interaction to regulate the CNS microenvironment in health and disease [2]. Following insults to the brain tissue, microglia undergo extensive transcriptional reprogramming, releasing a plethora of cytokines and inflammatory mediators. These secreted molecules act as messengers to facilitate communication between microglia and astrocytes [3]. Reactive astrocytes can regulate microglial functions in a similar manner (Fig. **1**).

Bidirectional communication between microglia and astrocytes determines their functional outcomes and regulates neuronal activity. Identifying putative molecules in theircellular crosstalk may provide better therapeutic options for many neurological disorders. To date, various molecules involved in interglial crosstalk have been identified and extensively reviewed by Jha *et al*. [2]. The current commentary briefly discusses the extant literature on the major molecules involved in reciprocal communication between microglia and astrocytes.

## 2 MICROGLIA-DERIVED MOLECULES REGULATING ASTROCYTES

Microglia-derived molecules play a definitive role in determining astrocytic function and fate in both health and disease. Several secreted molecules, including cytokines, neurotransmitters, growth factors, and microRNAs, are reportedly involved in the regulation of astrocyte activities. Recently, axon guidance molecules (AGMs) have emerged as key players in the regulation of microglia-astrocyte crosstalk. Microglia-derived molecules may confer beneficial functions or induce pathogenic activity in astrocytes in a context-dependent manner (Table **1**) [4-18].

Following changes in the brain microenvironment during pathological conditions, microglia activate inflammatory signaling pathways, including classical nuclear factor kappa B (NF-κB), mitogen-activated protein kinase, and phosphoinositide 3-kinase/protein kinase B, leading to increased secretion of pro-inflammatory mediators, such as interleukin 1 beta (IL-1β), tumor necrosis factor-alpha (TNF-α), and complement component 1q (C1q). Accumulating evidence has identified microglia-derived inflammatory mediators as triggers for inducing the neurotoxic phenotype of astrocytes in various disease states [5-8, 10, 12-14, 19]. Limiting microglial inflammatory signaling has been shown to inhibit astrocytic neurotoxicity.

A recent study identified microglia-derived AGMs' crucial role in potentiating astrocyte inflammatory activities in an experimental autoimmune encephalomyelitis (EAE) model [4]. The increased release of two AGMs, including semaphorin 4D (Sema4D) and Ephrin-B3, from microglia coincided with disease severity. Increased release of Sema4D and Ephrin-B3 from microglia activates astrocytic Plexin-B2 and EphB3 receptors, inducing neurotoxic astrocytes. Viral-mediated perturbation of these interaction pathways decreased disease severity in the EAE model, confirming the regulatory role of microglia in controlling astrocytic responses.

Rothhammer *et al.* provided an elegant example of the explicit control of astrocytic responses by microglia in EAE [11]. Vascular endothelial growth factor B (VEGF-B) released from inflammatory microglia induces neurotoxic astrocytes by interacting with its receptor, Fms-related receptor tyrosine kinase 1 (FLT1), in EAE, exacerbating ongoing neuroinflammatory signaling. Conversely, the release of transforming growth factor alpha (TGF-α) from microglia enhances the neuroprotective activities of astrocytes, leading to disease amelioration. Furthermore, VEGF-B-FLT1 upregulation and TGF-α-ErbB1 downregulation were found in patients with multiple sclerosis, corroborating findings observed in animal models.

Innate immune responses generated by brain-resident immune cells appear early in all neurodegenerative diseases, contributing to neurodegeneration. Previous reports have identified microglia as propagators of the inflammatory response induced by mutant protein aggregates in various neurodegenerative diseases. This notion has been confirmed in many *in-vitro* studies. The conditioned media from amyloid-beta (Aβ)-treated microglial cell cultures induced inflammatory signaling in astrocytes, while Aβ alone did not affect astrocyte reactivity [6, 10]. The transfer of conditioned media from α-synuclein preformed fibril-treated microglial cultures to astrocytes increased the transcripts of inflammatory genes [9]. In animal models of sporadic Parkinson’s disease (PD), the blockade of microglial inflammatory phenotype suppressed the induction of astrocytic neurotoxicity and resultant neurodegeneration [9]. In addition to classic inducers of inflammatory astrocytes, including IL-1α, IL-1β, TNF-α, and C1q, increased mitochondrial damage in microglial cells also triggered the neurotoxic reactivity of astrocytes in neurodegenerative diseases [15]. Increased mitochondrial fission in inflammatory microglia led to an extracellular release of dysfunctional mitochondria, which acted as an inflammatory signal, resulting in the induction of neurotoxic astrocytes [15].

Adequate glial responses play key roles in disease progression and mitigation. Microglia-derived molecules can also skew astrocytic reactivity toward beneficial roles and can aid in halting disease progression [18]. Insulin-like growth factor-1 is an immunomodulatory molecule released by microglia, which increases astrocyte migration and subsequent scar formation in spinal cord injury [16]. Scar-forming astrocytes are crucial for limiting the infiltration of peripheral immune cells and for dampening disease progression. Small vesicles enriched in miR-124 secreted from microglia also potentiated the beneficial function of astrocytes in an ischemic brain injury model. Initially regarded as AGM, plexins are now known to induce a plethora of immune functions in a context-dependent manner. Various ligands can activate plexin-mediated signaling, regulating cellular interactions. Contrasting findings have been recently reported regarding plexin-mediated immune effects in the CNS [20-22]. Zhou *et al.* found a protective role of microglial plexin-B2-signalling in regulating the physical interaction between microglia and astrocytes in an animal model of spinal cord injury [17]. Tissue repair following spinal cord injury depends on the proper arrangement of distinct cell types surrounding the injury sites, and microglial plexin-B2 aided in confining the territories of microglia and astrocytes. Plexin-B2 signaling in microglia regulates the spatial segregation of microglia and astrocytes around the injury site and determines the efficiency of wound healing [17].

Microglial cells are not homogenous populations and transit to many other phenotypes in a context-dependent manner. In addition, CNS is also home to numerous other types of myeloid cells with distinct localization, including perivascular, choroid plexus, and meningeal macrophages/dendritic cells [23]. Microglia are the major myeloid cells residing in the healthy brain parenchyma, hence named brain macrophages. In various neuroinflammatory conditions, bone marrow-derived peripheral myeloid cells are also found to infiltrate the brain tissue. Microglia share their surface markers with other myeloid cell types, making it difficult to identify brain-resident microglial cells in neuroinflammatory conditions [24, 25]. The heterogeneity and complexity of CNS myeloid cell dynamics linked to neuroinflammation require better tools to characterize the contribution of specific cell types to disease pathology. Further investigations are necessary to dissect the molecular, cellular, and phenotypic heterogeneity of myeloid cells in various neuroinflammatory diseases.

## 3 ASTROCYTE-DERIVED MOLECULES REGULATING MICROGLIAL FUNCTION IN HEALTH AND DISEASE

As discussed above, various studies have reported that microglia are upstream regulators of astrocyte functions. However, it is also important to consider the reciprocal regulation of microglia by astrocytes [2]. In this regard, various cytokines and chemokines, such as IL-1β, IL-10, IL-15, TNF-α, nitric oxide, and chemokine ligand 2 (CCL2) are also secreted by astrocytes, which act on microglia to regulate their functions [2, 26-31] (Table **2**).

Recent studies have further reported other molecules that play key roles in this nexus. Our laboratory and others have identified complement components, such as C3 and C8γ, which are primarily expressed by astrocytes and specifically bind to their receptors on microglial cells [37, 48, 52]. Activated astrocytes produce complement protein C3, which is cleaved to produce C3a and C3b. C3a then binds to the C3aR receptor on microglia, promoting their activation and C1q production, thereby causing local injury to neurons [38, 43, 53]. Based on this finding, various other groups have exploited this pathway to study astrocyte-microglia interactions in status epilepticus [40], hydrocephalus [39], depression [36], white matter injury [41], and prion disease [42].

Cathelicidins are a group of molecules with antimicrobial function and are part of the innate immune response [54]. Several studies have reported that mouse cathelicidin-related antimicrobial peptide (CRAMP) and its human homolog cathelicidin (LL-37) play an important role in a variety of neuroinflammatory conditions [55-61]. In a recent study, Bhusal *et al.* identified the role of CRAMP as a mediator of astrocyte-microglia crosstalk in EAE or multiple sclerosis [44]. At the molecular level, they reported that CRAMP, which is primarily expressed by astrocytes, potentiates the IFN-γ-induced STAT3 signaling pathway in microglia *via* formyl peptide receptors. In line with this, mice lacking cathelicidin showed a lower incidence of EAE with a reduction in T cell-mediated IFN-γ production [62]. Overall, these studies have unleashed the previously unidentified role of CRAMP in astrocyte-microglia communication.

Other mechanisms by which astrocytes regulate microglial functions during neuroinflammation involve the secretion of various proteins, such as fibronectin, Wnt5a, and secreted frizzled-related protein 1 (SFRP1) [32, 33]. In particular, SFRP1 expressed by astrocytes during neuroinflammation led to the transcription of the downstream targets of hypoxia-inducible factor and NF-κB in microglial cells, promoting their activation [33]. Other studies have found that NF-κB activation in astrocytes under various conditions causes the expression and/or release of CCL2, Wnt5a, and granulocyte-macrophage colony-stimulating factor (GM-CSF), which leads to the activation and expansion of microglia cells [34, 35, 63]. Specifically, Baumann and colleagues uncovered a significant role of the time-dependent regulation of NF-κB in astrocytes, which ultimately determines the fate of microglia to have either protective or detrimental function. In their study, IKK2/NF‐κB was overexpressed in astrocytes of the SOD1 (G39A) mice, an amyotrophic lateral sclerosis (ALS) animal model. To their surprise, SOD1/IKK2 mice displayed delayed onset of the disease but with increased severity as the disease progressed. They have reported that NF‐κB activation in astrocytes led to significant upregulation of Wnt5a, which acted on microglia to increase their proliferation, delaying the presymptomatic phase of ALS, but greatly increasing the severity of the symptomatic stage [34, 64]. Furthermore, another study has shown that secretome obtained from cortical astrocytes of mSOD1 mice upregulates the expression of *iNOS* and *Tnf* genes in microglia [65]. These findings suggest that astrocyte-microglia crosstalk might regulate the overall inflammatory responses in neurodegenerative diseases such as ALS.

Small extracellular vesicles (sEVs) released from cells play a key role in cellular communication by transporting RNA, proteins, and bioactive lipids between cells [66]. Astrocytes can produce and export these sEV containing proteins and RNAs that affect different functions in target cells [67]. In the study by Rong *et al*., astrocytes were found to release sEVs encapsulating CCL2 during spinal cord injury [27]. Immunostaining analysis showed that CCR2, the main receptor for CCL2, was expressed predominantly by microglia and neuronal cells. They found that astrocytic sEVs containing CCL2 were transported to microglia and neuronal cells; CCL2 binding to CCR2 resulted in microglial activation. The activated microglia released IL-1β that further acted on the neurons aggravating neuronal apoptosis [27]. These results support that sEVs can be a potential mediator of astrocyte-microglia communication during neuroinflammation.

The astrocytic regulation of microglial function has also been implicated in homeostatic and anti-inflammatory conditions. Vainchtein *et al.* found that IL-33 produced by astrocytes regulated microglial synapse engulfment in the developing brain, suggesting the important role of interglial crosstalk in the sculpting of neural synapses and shaping of neural circuits [47]. Another group supported this finding; they found that astrocyte-microglia communication mediated by the IL-33-suppression of tumorigenicity (ST) 2-AKT pathway helped microglia for metabolic adaptation and phagocytic function during early development [46]. McAlpine *et al.* recently investigated the role of astrocyte-microglia interaction in the clearance of Aβ and the prevention of cognitive decline in the mouse model of Alzheimer’s disease (AD). They found that astrocyte-specific IL-3 acts on microglial IL-3Rα receptors endowing them with enhanced motility and the capacity to clear amyloid plaques [45]. Recently, Kim *et al.* found that C8γ, primarily expressed by astrocytes under AD and other neuroinflammatory conditions, interacts with the sphingosine-1-phosphate (S1P) receptor of microglia to antagonize the pro-inflammatory action of S1P [48]. They showed that shRNA-mediated knockdown of C8γ inhibits glial hyperactivation, neuroinflammation, and cognitive decline in acute and chronic animal models of AD [48]. This was an interesting observation, as the majority of complement components are thought to be proinflammatory in nature.

Exosomes are a well-characterized subtype of EVs [68, 69]. In the study by Jing *et al.*, astrocytes were found to release exosomes containing miR-137 under the oxygen-glucose deprivation/reperfusion conditions; these astrocyte-derived exosomes were taken up by microglia, causing their transformation to an anti-inflammatory M2 phenotype [49]. They further demonstrated that upregulation of miR-137 could significantly inhibit inositol polyphosphate 4-phosphatase expression, negatively regulating the PI3K/Akt pathway involved in cell survival and proliferation [49]. In another study, Long *et al.* have shown that astrocyte-derived exosomes containing miR-873a-5p attenuated microglia-mediated neuroinflammation and improved neurological deficits following traumatic brain injury by inhibiting the NF-κB signaling pathway [50]. Similarly, other studies have also proved the protective effects of astrocyte-derived exosomes in different neuroinflammatory conditions [70, 71]. Indeed, releasing exosomes may be one way for astrocytes to communicate with neighboring cells like microglia.

Apart from secreted molecules, state-of-the-art imaging techniques have identified the appearance of novel physical structures called nanotubes, forming direct physical contact between astrocytes and microglia for effective communication [51, 72]. These nanotubes allow for the intercellular transfer of aggregated proteins such as α-synuclein and Aβ from astrocytes to microglia, which are then broken down and removed from the culture [51]. The impairment of direct contact between astrocytes and microglia may further hamper the branching and migration of microglia [73]. Taken together, there appears to be an intimate astrocyte-microglia interaction at the functional and physical levels in both homeostatic and pathological states, thereby allowing them to govern and regulate each other’s functions.

## 4 PERSPECTIVE AND FUTURE DIRECTIONS

The functions of glial cells are becoming more apparent with the identification of molecules they release. It has recently been shown that glial cells use these secretory molecules to functionally interact with other cell types. A better understanding of the complex microglia-astrocyte crosstalk could lead to the discovery of new diagnostic biomarkers and therapeutic targets for neuroinflammation and related disorders. As we summarized previously [2], most studies have focused on understanding well-known inflammatory mediators of cellular communication, such as cytokines, chemokines, and acute-phase proteins, which act through receptors in other cell types. Recently, other modes of communication involving enzymes, gliotransmitters, mitochondria, exosomes, AGMs, and cell-cell tunneling systems have been identified. Henceforth, we have begun to appreciate the importance of this microglia-astrocyte nexus. The development of advanced imaging systems, single-cell analysis techniques, transgenic animal models with cell-specific promoters, and elegant mouse genetics have further aided in expanding the understanding of this complex system.

Interglial crosstalk and targeted therapies based on this crosstalk are still in their infancy, and several issues remain to be further examined. One such difficulty is the presence of regional heterogeneity in the microglia and astrocytes under diverse conditions. This has led to the need to map these newly found glial molecules *in vivo* in real-time and to comprehend their relative roles within tissues in both homeostatic and pathological states.

Both microglia and astrocytes release TNF-α and IL-1β, among others, to regulate each other’s reactive states, which may ultimately lead to neurotoxicity [3, 29]. These proinflammatory cytokines are well known to induce apoptotic damage to brain cells, including neurons and oligodendrocytes [74]. Under these circumstances, microglia and astrocytes might also be susceptible to the toxic effects of proinflammatory mediators. The studies on this aspect are limited. However, the death-resistant nature of murine and human microglia and astrocytes, compared to neurons, has been reported in various *in-vitro* studies [75-78]. In addition, under *in-vivo* conditions, neuronal damage precedes glial damage, implying that glial cells can withstand the damage even under a neuroinflammatory environment [79].

Another main challenge for glia interaction research is to handle and study a large number of new molecules being analyzed. This will require the combined effort of experts from several disciplines to pinpoint molecules and study their potential in neuroinflammation and neurodegenerative diseases. Furthermore, in many cases, the results have been oversimplified, and the use of non-physiological concentrations of glial molecules/proteins may provide some artifactual insights into the crosstalk. As a result, it is difficult to interpret the usefulness of the identified crosstalk. Moreover, a substantial translational gap between animal studies and clinical implications still exists. Nevertheless, exploration of glial molecules/proteins in human post-mortem tissues, cerebrospinal fluid and blood samples, as well as employment of human iPSC-derived glial cells [80, 81] and minimally invasive imaging techniques, can provide a functional readout for specific cells or molecules from a clinical perspective [80]. We believe that the clinical harnessing of glial molecules to diagnose and treat neurodegenerative diseases requires more basic research to better understand the molecular mechanisms underlying the crosstalk between them.

## Figures and Tables

**Table 1:** Microglia-secreted molecules regulating the fate of astrocytes.

**Phenotype**	**Category**	**Molecules**	**Implications**	**References**
Pro-inflammatory/ neurotoxic	AGMs	EphrinB3 Sema4D	**🡡**Proinflammatory cytokines **🡡**EAE-associated pathology **🡡**NOS2 and IL-1β	[4]
	Cytokines	IL-18	**🡡**NF-κB signaling **🡡**Migraine pain	[5]
		IL-1β	**🡡**NF-κB signaling	[6]
		TNF-α	**🡡**NF-κB signaling **🡡**Post-surgical pain **🡡**Epileptogenesis **🡡**Disease burden in AD and PD	[6-9]
		IL-1α	**🡡**Post-surgical pain **🡡**Epileptogenesis **🡡**Neurodegeneration in PD	[7-9]
	Complement	C1q	**🡡**Post-surgical pain **🡡**Neurodegeneration in PD	[7, 9, 10]
	Growth factor	VEGF-B	**🡡**EAE-associated pathology	[11]
		TGF-β1	**🡡**Scar-associated pathology	[12]
	Vesicles/exosomes containing miRNA	miR-145-5p	**🡣**Astrocyte proliferation	[13]
	Pro-inflammatory mediators	ROS	**🡡**Pain hypersensitivity	[14]
	Organelle	Damaged mitochondria	**🡡**Neurodegeneration in ALS, AD, and HD	[15]
Anti-inflammatory/ neuroprotective	Growth factor	IGF-1	**🡡**Axonal regeneration and functional recovery	[16]
		TGF-α	**🡣**EAE pathology	[11]
	AGMs	Plexin-B2	**🡡**Wound healing	[17]
	Vesicles containing miRNA	miR-124	**🡡**Recovery after stroke	[18]

**Abbreviations**: AGMs, axon guidance molecules; Sema4D, semaphorin-4D; IL, interleukin; NF-κB, nuclear factor kappa-light-chain-enhancer of activated B cells; TNF, tumor necrosis factor; TGF, transforming growth factor; C1q, complement component 1 q; NOS, nitric oxide synthase; VEGF-B, vascular growth factor beta; miR, microRNA; IGF-1, insulin-like growth factor 1; EAE, experimental autoimmune encephalomyelitis; AD, Alzheimer’s disease; ALS, amyotrophic lateral sclerosis; HD, Huntington’s disease; PD, Parkinson’s disease.

**Table 2:** Astrocyte-derived molecules regulating microglial function in health and disease.

**Phenotype**	**Category**	**Molecules**	**Implications**	**References**
Pro-inflammatory/ neurotoxic	ECM	Fibronectin	**🡡** TNF-α production **🡡** Disease burden in SCI	[32]
	Growth modulators	SFRP1	**🡡**HIF, NF-κB - Sustain a chronic inflammation in EAE	[33]
		Wnt5a	**🡡** CD68 **🡡** Microglia proliferation **🡡** Disease burden in ALS	[34]
	Cytokines	IL-15	**🡡** CD86, IL-1β, and TNF-α production **🡡** Brain injury following ICH	[30]
		IL-1β	**🡡** M1 polarization **🡡** Neuroinflammation in 1,2-DCE-intoxicated mice	[31]
		TNF-α	**🡡** M1 Polarization **🡡** Neuroinflammation in 1,2-DCE-intoxicated mice **🡡** Disruption in risk assessment behavior	[29, 31]
		CCL2	**🡡** Microglia activation **🡡** IL-1β production **🡡** Neuronal apoptosis **🡡** Disease burden in SCI **🡡** Neuroinflammation in LPS-injected mice	[27, 35]
		GM-CSF	**🡡** Microglia activation **🡡** Neuroinflammation in LPS-injected mice	[35]
	Complement	C3	**🡡**Neuroinflammation **🡡**Disease pathology in neuromyelitis optica, Alzheimer’s disease, epilepsy, depression, hydrocephalus, white matter injury, and prion disease	[36-43]
	Cathelicidins	CRAMP/ LL-37	**🡡**Neuroinflammation **🡡**Disease burden in EAE	[44]
	Gliotransmitter	Glutamate	**🡡**Microglia expansion and reactivity **🡡**Neuroinflammation **🡡**Disruption in risk assessment behavior	[29]
-	Pro-inflammatory mediators	NO	**🡡** M1 polarization **🡡** Neuroinflammation in 1,2-DCE-intoxicated mice	[31]
Anti-inflammatory/ neuroprotective	Growth modulators	Wnt5a	**🡡** IL-6, CD206, CD163 **🡡** Microglia proliferation - Disease onset delayed in ALS	[34]
	Cytokines	IL-3	**🡡** Motility of microglia **🡡** Clearance of Aβ and tau aggregates -Restrict AD progression and cognitive decline	[45]
		IL-33	**🡡** Synapse engulfment -Improved synaptic pruning -Supports microglial metabolic adaptation and phagocytic function	[46, 47]
	Complement	C8γ	-Antagonizes the pro-inflammatory action of S1P in microglia **🡣**Inflammation -Therapeutic containment of neuroinflammation in AD	[48]
	Vesicles/exosomes containing miRNA	miR-137	-Promote M2 polarization of microglia -Increase cell viability and attenuate apoptosis following OGD/R injury	[49]
		miR-873a-5p	**🡣** NF-κB signaling **🡣** Inflammation -Attenuate neurological deficits following TBI	[50]
	Nanotubes	-	**🡡** Degradation of αSYN and Aβ aggregates	[51]

**Abbreviations**: ECM, extracellular matrix; SCI, spinal cord injury; SFRP1, secreted frizzled-related protein 1; Wnt5a, wingless-type MMTV integration site family member 5a; IL, interleukin; ICH, intracerebral hemorrhage; CCL2, chemokine (C-C motif) ligand 2; GM-CSF, granulocyte-macrophage colony-stimulating factor; TNF, tumor necrosis factor; NO, nitric oxide; HIF, hypoxia-inducible factor; NF-κB, nuclear factor kappa-light-chain-enhancer of activated B cells; C3, complement component 3; C8γ, complement component 8 gamma; CRAMP, cathelicidin-related antimicrobial peptide; LL-37, leucine leucine 37; CD, cluster of differentiation; S1P, sphingosine-1-phosphate; ALS, amyotrophic lateral sclerosis; αSYN, alpha-synuclein; Aβ, amyloid beta; TBI, traumatic brain injury; OGD/R, oxygen-glucose deprivation/reperfusion; AD, Alzheimer’s disease; DCE, dichloroethane; EAE, experimental autoimmune encephalomyelitis; LPS, lipopolysaccharide.

## LIST OF ABBREVIATIONS

AGMs: Axon Guidance Molecules
C1q: Complement Component 1 q
C3: Complement Component 3
C8γ: Complement Component 8 Gamma
CCL2: Chemokine (C-C motif) Ligand 2
CNS: Central Nervous System
CRAMP: Cathelicidin-related Antimicrobial Peptide
CSF: Cerebrospinal Fluid
EAE: Experimental Autoimmune Encephalomyelitis
EphB3: Ephrin B3 Receptor
ErbB1: Erb-B1 Receptor Tyrosine Kinase
FLT1: Fms-related Receptor Tyrosine Kinase 1
GM-CSF: Granulocyte-macrophage Colony-stimulating Factor
HIF: Hypoxia-inducible Factor
IGF: Insulin-like Growth Factor
IL: Interleukin
iNOS: Inducible Nitric Oxide Synthase
LL-37: Leucine Leucine-37
NF-κB: Nuclear Factor Kappa-light-chain-enhancer of Activated B Cells
NO: Nitric Oxide
ROS: Reactive Oxygen Species
S1P: Sphingosine-1-Phosphate
Sema4D: Semaphorin-4D
sEVs: Small Extracellular Vesicles
SFRP1: Secreted Frizzled-related Protein 1
ST: Suppression of Tumorigenicity
TGF: Transforming Growth Factor
TNF: Tumor Necrosis Factor
VEGF: Vascular Endothelial Growth Factor
Wnt5a: Wingless-type MMTV Integration Site Family Member 5a
